# Biophysics of Phase Separation of Disordered Proteins Is Governed by Balance between Short- And Long-Range Interactions

**DOI:** 10.1021/acs.jpcb.0c09975

**Published:** 2021-02-25

**Authors:** Milan Kumar Hazra, Yaakov Levy

**Affiliations:** Department of Structural Biology, Weizmann Institute of Science, Rehovot 76100, Israel

## Abstract

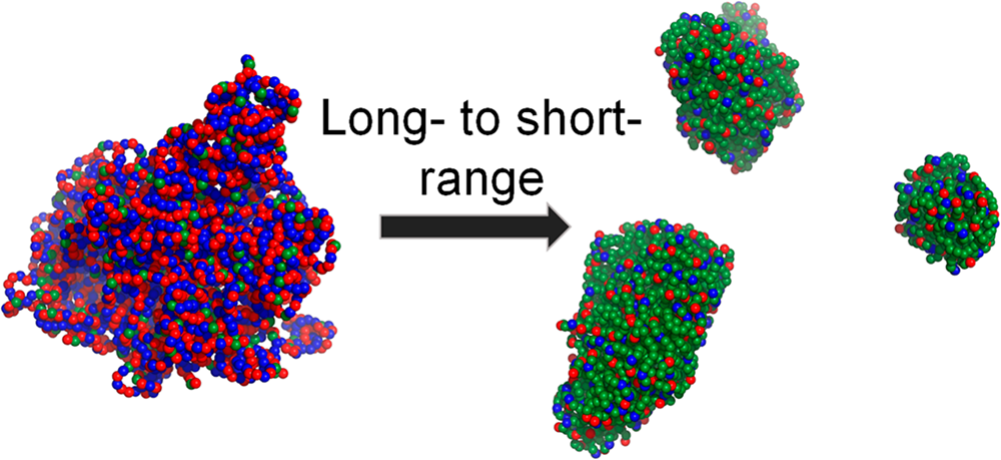

Intrinsically disordered proteins play a crucial role in cellular phase separation, yet the diverse molecular forces driving phase separation are not fully understood. It is of utmost importance to understand how peptide sequence, and particularly the balance between the peptides’ short- and long-range interactions with other peptides, may affect the stability, structure, and dynamics of liquid–liquid phase separation in protein condensates. Here, using coarse-grained molecular dynamics simulations, we studied the liquid properties of the condensate in a series of polymers in which the ratio of short-range dispersion interactions to long-range electrostatic interactions varied. As the fraction of mutations that participate in short-range interactions increases at the expense of long-range electrostatic interactions, a significant decrease in the critical temperature of phase separation is observed. Nevertheless, sequences with a high fraction of short-range interactions exhibit stabilization, which suggests compensation for the loss of long-range electrostatic interactions. Decreased condensate stability is coupled with decreased translational diffusion of the polymers in the condensate, which may result in the loss of liquid characteristics in the presence of a high fraction of uncharged residues. The effect of exchanging long-range electrostatic interactions for short-range interactions can be explained by the kinetics of breaking intermolecular contacts with neighboring polymers and the kinetics of intramolecular fluctuations. While both time scales are coupled and increase as electrostatic interactions are lost, for sequences that are dominated by short-range interactions, the kinetics of intermolecular contact breakage significantly slows down. Our study supports the contention that different types of interactions can maintain protein condensates, however, long-range electrostatic interactions enhance its liquid-like behavior.

## Introduction

Liquid–liquid phase separation (LLPS) mediates several fundamental cellular activities such as signaling,^1^ RNA metabolism,^2^ and stress adaptation^3^ to name a few. Recent advances have identified LLPS as the primary mechanism^4−6^ for the formation of common membraneless organelles, such as stress granules and nucleoli, as well as heterochromatin assembly,^6,7^ centrosomes,^8^ presynaptic^9,10^ and prosynaptic densities,^11^ and membrane receptor clusters.^12^ Intrinsically disordered proteins (IDP), which have numerous different chain conformations, play a key role in biological condensates.^13,14^ The highly fluctuating and elongated conformations of IDPs are known to govern the protein network in such condensates.^15^ This may lead to phase separation at concentrations lower than are needed for folded proteins.^16^

Various aspects of LLPS are still under intensive investigation, including the contribution of LLPS to proper cellular functioning and to disease states, as well as the microscopic structure and stability of the dense phase and its relation to liquid-like features. Different biological condensates have very different substructures that are relevant to their functionalization. Organelles formed by proteins, such as the DNA-binding protein FUS, are known to be liquid-like in nature,^17^ whereas the condensate of another DNA-binding protein, TDP-43, forms a gel-like assembly.^18^ Even stress granule proteins form liquid-like polymer-rich condensate droplets (called coacervates), which in turn mature and solidify.^19^

The liquid-like or gel-like character of the dense phase primarily originates from the nature of the specific type of interactions in the condensate. Using bioinformatics tools, computational approaches, and in vitro experiments to elucidate the molecular driving force of LLPS, several recent studies identified that different types of interactions contribute to the enthalpy mediated stabilization of complex coacervation by IDPs.^20−22^ Major molecular contributions that govern the condensate properties have been assigned to charge–charge, cation−π, and π–π interactions.^23−26^ These studies showed that the composition as well as the organization^14,27−30^ of charged and aromatic/hydrophobic residues can strongly affect LLPS. These molecular interactions entropically stabilize the condensate, and the balance between their enthalpy and entropy components^31,32^ dictates the nature of the condensates. For example, direct evidence from NMR relaxation and diffusion data shows that elastin-like hydrophobic domains form an entropy-mediated simple condensate.^33^ For the human Tau protein, rapid large amplitude torsional fluctuations in extended chains have been observed in picosecond time-resolved fluorescent depolarization measurements, which indicate the dynamic liquid-like nature of the dense phase.^13^ A detailed understanding of the microscopic structure, stability, and dynamics is still required in order to correlate the nature of the condensate to the specific kind of interaction involved.

Theoretical formulations as well as molecular dynamics simulations, using atomistic and coarse-grained representations, have joined the effort to characterize the phase behavior and microscopic structural features of polymers in dense coacervates.^28,29,34−45^ These studies, though missing explicit respresenation of the solvent or of counterions, capture basic structural aspects and phase behavior of model polyampholyte systems or natural proteins that participate in LLPS. Recent advances reveal that the presence of sequence-specific re-entrant phase behavior (where the density of the condensate phase increases, reaches a maximum, and then decreases) plays an important role in the dissolution and dynamic substructure formation of biological assemblies.^27,46,47^ Such re-entrant phase behavior is also present in proteins,^27,46−51^ patchy particles,^52^ and network forming systems.^53,54^ In protein solutions, such phase behavior is observed in response to varying temperature or salt concentration.^48−51^ Field theory models have also been invoked to predict the phase behavior of protein condensates.^55^ Several studies use the “stickers and spacer model”, which has shown significant promise in maintaining the fluidity of polymers in the dense phase.^23^ In our previous work, using coarse-grained molecular dynamics simulations, we showed how the sequence charge pattern affects the microscopic structure and dynamics of polyampholyte condensates.^14^

In the current study, we address the question of how short-range site-specific interaction motifs in IDPs can affect the stability and dynamics of their condensates. Starting from a fully charged polyampholyte sequence that serves as a reference system, we gradually mutated the charged residues with residues participating in short-range dispersion interactions. Accordingly, the long-range electrostatic interactions in the dense phase were progressively replaced by short-range dispersion interactions. Thus, we aimed to understand how the liquid-like features of the dense phase and its stability are related to the proportions of these two types of common IDP interactions.

## Methods

### Studied Systems

**Figure 1:**
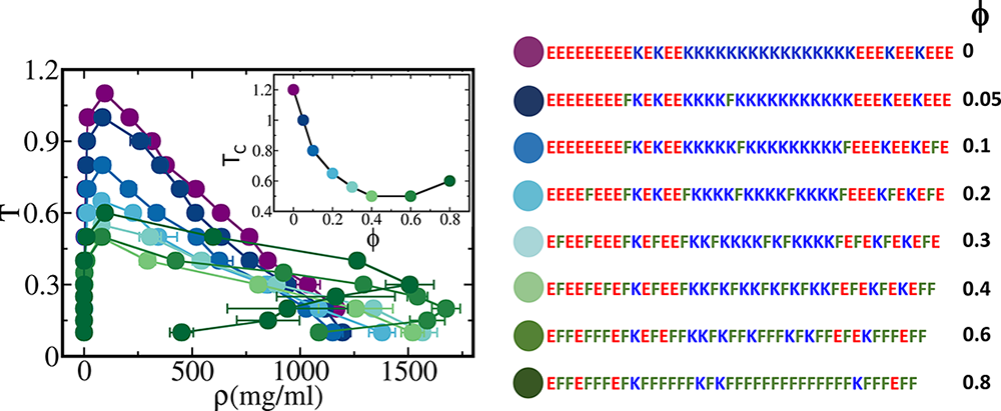
Phase diagram and criticality. Temperature–density phase diagram of eight designed 40-residue sequences whose hydrophobicity fraction, ϕ, is 0–0.8. The peptide with ϕ = 0 corresponds to a polyampholyte whose charges are organized to yield a charge mixing value of κ = 0.55. The sequences and their corresponding ϕ parameter values are shown on the right. Residues that are marked in blue and red correspond to positively and negatively charged residues, respectively (and participate in long-range interactions between themselves, either intra- or intermolecularly), whereas those marked in green correspond to hydrophobic residues (and participate in short-range interactions between themselves, either intra- or intermolecularly). The critical temperature (*T*_c_) was evaluated from the following relation of the universal scaling of density near the critical point, . The inset shows the relationship between *T*_c_ and the ϕ parameter for the eight sequences. The coloring in the plots follows the color scheme of the sequences.

To investigate the stability and dynamics of the condensates formed by IDPs, we employed a coarse-grained molecular dynamics simulation that has been applied recently to study LLPS in polyampholytes.^56^ The sequences studied here comprised 40 residues, each modeled by a single bead per residue. The beads were either charged or neutral. The neutral beads, which are involved in short-range dispersion interactions and represent hydrophobic residues, were introduced by mutating an equal number of positively and negatively charged residues, while retaining an over net charge of zero on the IDP. The peptide without hydrophobic residues (ϕ = 0) included 20 positively and 20 negatively charged residues that were organized to obtain a charge mixing value of κ = 0.55. This peptide, which served as a reference peptide, was selected because its midrange κ value was previously shown to correspond to the intermediate liquid properties of the condensate. This fully charged peptide was mutated to generate seven additional peptides with 2–32 hydrophobic residues (i.e., ϕ = 5–80%). The exact sequences of the studied peptides are shown in Figure 1.

### Coarse-Grained Model

The potential energy function of the coarse-grained model consists of several terms: the bonded, angular, and electrostatic interactions among all the charged beads, and short-range dispersion interaction among all pairs of neutral beads. In addition, an excluded volume potential is applied between all bead pairs. The bonded and angular interactions are modeled with a harmonic potential. As intrinsically disordered proteins have a high conformational flexibility, which is important in the formation of biological network-based condensates, polymers are modeled as completely flexible without any dihedral constraint.

**Figure 2:**
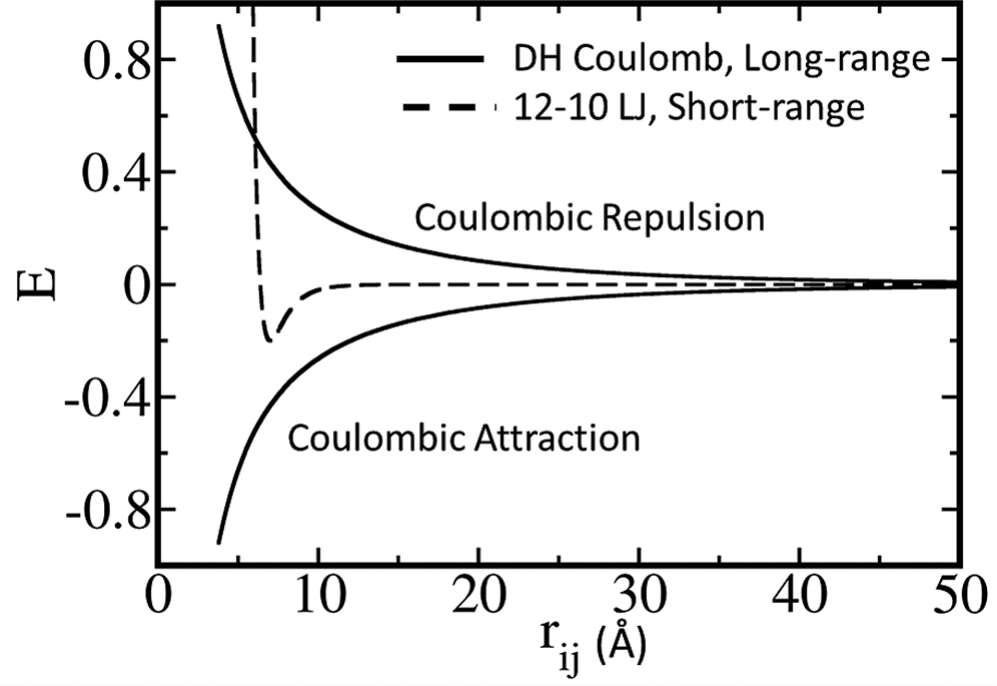
Comparison between the short- and long-range potentials. The 10–12 Lennard-Jones (LJ) potential that represents the short-range interactions and the Debye–Huckel (DH) Coulombic potential that represnts the long-range interactions are plotted as a function of the pairwise distance, *r*_*ij*_. The Lennard-Jones is plotted for an optimal distance of 7 Å. The Debye–Huckel is plotted for both repulsive and attractive cases for a salt concentration of 0.02 M.

The electrostatic interactions are modeled with the Debye–Hückel potential^56,57^, where *q*_*i*_ and *q*_*j*_ denote the charge of the *i*^th^ and *j*^th^ bead, *r*_*ij*_ denotes the interbead distance, ε is the solvent dielectric constant, and *K*_coulomb_ = 4πε_0_ = 332 kcal/mol. The term *B*(κ) is dependent on salt concentration and the radius (*a*) of ions produced by the dissociation of the salt and is given by . According to Debye–Hückel theory, the range of electrostatic interactions of an ion is of the order of κ^–1^, which is called the Debye screening length. The Poisson–Boltzmann equation leads to the following relation of κ to ionic strength, , where *N*_A_ is the Avogadro number, *e* is the charge of an electron, *ρ*_A_ is the solvent density, *I* denotes the ionic strength of the medium, *k*_B_ is the Boltzmann constant, and *T* is the temperature. The neutral residues interact with each other with a short-range dispersion potential modeled by the Lennard-Jones term, , where σ_*ij*_ denotes the optimal distance between beads *i* and *j* that are in contact with each other, and σ_*ij*_ = 7 Å. The term ε is the strength of the short-range interaction and its value was selected to represent realistic IDP behavior. To achieve that, we compared the radius of gyration (*R*_g_) values of several IDPs estimated from simulating the coarse-grained model with ε values that differ from their experimental values (see Supporting Information). A value of ε = 0.2 shows the best correlation between the calculated and measured *R*_g_ (see Figure S1). Unless stated otherwise, a value of ε = 0.2 was used in all the simulations whose results are presented here. To compare the short- and long-range interactions, we plot the Debye–Huckel electrostatics interaction energy at a salt concentration of 0.02 M and short-range 12–10 LJ potential with ε = 0.2 (Figures 2 and S2). In addition to these two specific kinds of interactions, and in order to avoid overlap, all the beads interact with each other by a short-range repulsion of the form  with *σ*_*ij*_ = 4 Å.

The starting structure was built by randomly placing 100 polymers in a box with dimensions 300 × 300 × 300 Å, where each polymer consisted of 40 residues. The simulations were performed for a total of 10^7^ steps with salt concentration 0.02 M (we note that due to the coarse-grained representation, and given that the charge is placed in the backbone itself, the salt concentration used in the simulations represents a higher effective salt concentration than its nominal value). The convergence of the coarse-grained simulations was tested by following the time evolution of the density of the dense phase and the mean squared displacements for several independent copies of simulations (Figures S3 and S4). The effect of the system size was examined by calculating the phase diagram for 50, 150, and 200 chains (Figure S5). In the simulations, the dielectric constant was 80 and temperatures were maintained below the critical temperature of the sequence of interest. At each temperature, multiple trajectories were simulated by solving the Langevin equation to achieve proper averaging. To initiate the simulation, a random unfolded conformation was used for each polyampholyte in the simulation box. A clustering algorithm was used to identify the largest condensate and its component polymers at each time step.

In addition to the effect of increasing the fraction of short-range (hydrophobic) interaction motifs (ϕ) in a sequence, we also investigated the effect of varying the short-range interaction strength (ε) on the stability and dynamics of the condensate. The effect of the ε value was examined for the sequences with ϕ = 0.1, 0.2, 0.4, and 0.6. Variation in the value of ε may represent mutations to residues with different hydrophobicity strengths. For each of the selected sequences with a specific ϕ, simulations were performed at short-range interaction strengths of ε = 0.0, 0.2, 0.3, 0.4, and 0.5 in order to explore variations in the structure, stability, and dynamics of the condensate phase. The presented results refer to ϕ = 0.2, unless otherwise stated.

### Structural Analysis

The structure of the IDPs in the condensate was quantified by their *R*_g_ values and was compared to the corresponding *R*_g_ values in the bulk. This comparison was made for a series of IDPs having different ϕ values and at different temperatures. In addition to the conformations of the IDP that comprise the condensate, the overall shape of the condensate may also depend on ϕ and *T*. To quantify the shape of the condensate, we estimated shape anisotropy by calculating the following parameter:
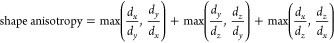
where *d*_*x*_, *d*_*y*_, and *d*_*z*_ measure the largest diameters of the largest cluster in the *X*, *Y*, and *Z* dimensions, respectively. Following this measure, a spherically shaped condensate has a shape anisotropy value of 3, while more elongated condensate shapes acquire values larger than 3.

### Dynamics Analysis

The liquid property of the condense phase was described by the diffusion coefficient, estimated by the slope of the mean squared displacements (MSD) of the center of mass (COM) of each polymer in the dense phase when plotted versus time as MSD = 6 Dt. Furthermore, two additional dynamic properties of the polymers in the droplet were followed: the time scale for the exchange of interactions with a neighboring polymer in the condensate (τ_interchain_) and the time scale for intramolecular conformational change in the polymers that comprise the condensate (τ_intrachain_). The τ_interchain_ values were estimated by calculating a time-correlation function for the breaking of intermolecular interactions, which was defined as the relaxation time in the following correlation function: , where *h*(*t*) is a step function defined as *h*(*t*) = 1 when two polymers are nearest neighbors and, otherwise, *h*(*t*) = 0. In the dense phase, if at least five amino acid residues from each of two polymer chains lay within a distance of 10 Å from each other, we considered the polymers to be nearest neighbor. The intrachain dynamics in the dense phase was probed by the relaxation of the time-correlation function of the polymers’ end-to-end distance fluctuations (also known as their internal dynamics), which is defined as follows: , where *di*(*t*) represents the end-to-end distance of the *i*th polymer in the dense phase.

## Results and Discussion

Replacing charged residues with hydrophobic or aromatic residues is expected to affect the stability of the condensate formed via LLPS, as these residues are involved in different types of interactions. Whereas charged residues participate in electrostatic interactions that are long-range in nature, hydrophobic and aromatic residues participate in dispersion interactions, such as π–π interactions, that are much shorter range. Changing the number of hydrophobic or aromatic residues at the expense of charged residues is therefore expected to affect the biophysical characteristics of the condensates formed by these peptides. To quantify how the degree of hydrophobicity in the peptides affects the stability and liquid properties of condensates, we studied a series of eight 40-residue peptides with sequences that include up to 80% hydrophobic/aromatic residues. Coarse-grained molecular dynamics simulations were performed for each of the eight peptides at various temperatures. Constructing the phase diagram for the LLPS of each peptide is essential to identify the critical temperature, *T*_c_, of its phase separation.

### Stability and Energetics of the Dense Phase

Temperature–density phase diagrams of the eight sequences (having hydrophobic residue fractions, ϕ, in the range 0–0.8) highlight the *T*_c_ of each system and, hence, their relative stability. The critical temperatures was evaluated by fitting each curve to the following equation: , where *ρ*_dense phase_ and *ρ*_dilute phase_ denote the polymer bead density in the dense phase and in the dilute phase, respectively, and β is a critical exponent that, following other studies, was set to 0.325.^14^ As ϕ increases, *T*_c_ is observed to drop up to two-fold (Figure 1, inset), indicating that the stability of the dense phase decreases as the number of hydrophobic residues increases. The decrease in *T*_c_ with increasing ϕ is not monotonic, and a moderate increase in *T*_c_ is even observed for very high ϕ. Accordingly, replacement of long-range interactions with short-range interactions reduces the stability of the condensate up to a certain value, beyond which a reverse effect is observed in which the condensate gains greater stability.

**Figure 3:**
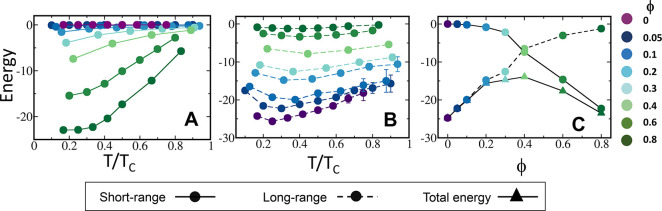
Enthalpic stability of the dense phase. Interchain (A) short-range interaction energy (circles with solid lines) and (B) long-range energy (circles with dashed lines) in the dense phase for the eight designed sequences as a function of temperature scaled with respect to the critical temperature (*T*/*T*_c_) of each sequence. The short- and long-range interactions correspond to the dispersion interactions between the hydrophobic residues and the electrostatic interactions between charged residues, respectively. (C) Trend in total interchain energy (i.e., the sum of the short- and long-range energies, shown with triangles) in the dense phase as a function of ϕ at *T* = 0.3*T*_c_. In all these simulations, ε = 0.2 for the short-range dispersion interactions.

To understand the nonlinear dependence of *T*_c_ on ϕ, we measured the contributions of interchain electrostatic and hydrophobic energy to the total energy of the condensate for the eight sequences at different temperatures (Figure 3A,B). As expected, the contribution of the short-range LJ interaction increases as ϕ increases from 0 to 0.8 (Figure 3A). This is coupled with a decrease in the strength of the electrostatic energy as φ increases. For a given value of *T*/*T*_c_, as the ϕ parameter increases from 0 (purple line) to 0.8 (olive line), the absolute value of the energy of the long-range electrostatic interactions decreases (Figure 3B). To correlate the changes in the electrostatic and hydrophobic energies with the stability of the condensate, it is necessary to quantify the trade-off between the short- and long-range interactions. Figure 3C shows the total intermolecular energy (i.e., *E*_total_ = *E*_short-range_ + *E*_long-range_) as a function of ϕ for *T* = 0.3*T*_c_ to reveal nonmonotonic behavior (see also Figure S6). The total energy is less favorable (i.e., has more positive values) as ϕ increases from 0 to 0.4, but changes to a trend of more favorable energies (i.e., more negative values) as ϕ increases from 0.4 to 0.8. It is likely that when only a few charged residues are mutated with hydrophobic residues (i.e., ϕ < 0.4), electrostatic interactions are diminished because fewer residues can participate in long-range interactions. However, when the sequences are enriched with hydrophobic residues (i.e., ϕ > 0.4), in addition to the smaller contribution of long-range electrostatic interactions, many possibilities arise for the formation of short-range interactions, resulting in an overall stabilization of the condensate compared with sequences with lower values of ϕ. The favorable intermolecular interactions at low and high ϕ, whereas medium ϕ shows weaker interactions, explain the nonmonotonic dependence of *T*_c_ on ϕ (Figure 1).

### Re-Entrant Phase Behavior for Highly Hydrophobic Peptides

Sequences with ϕ > 0.4 (Figure 1, main graph, dark green lines) show significant re-entrant phase behavior at low temperatures, such that the density of the condensate increases, reaches a maximum, and there-after decreases with decreasing temperature. Similar re-entrant behavior has been observed recently both computationally^27,52,58^ and experimentally^47,59,60^ for other systems. The decrease in density with decreasing temperature is linked with these systems being more structured because of the existence of larger sticky patches (e.g., larger cluster of hydrophobic residues).^27^

**Figure 4:**
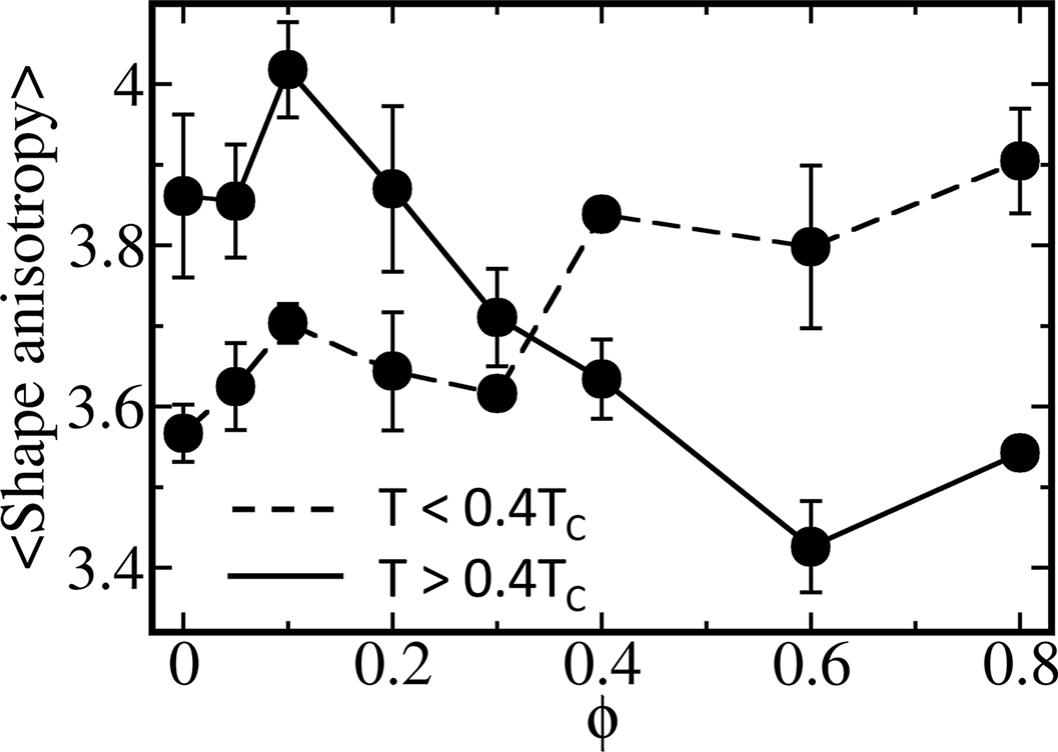
Shape of the condensates. Variation of the shape anisotropy parameter of the condensate with the hydrophobic content, ϕ, of the eight studied sequences. A value of 3 for the shape anisotropy refers to an ideal sphere, with values >3 referring to a more elongated shape. The mean anistropy parameter for each sequence was estimated for two cases: low (dashed line) or high (solid line) temperatures. The mean anisotropy parameters at low and high temperatures were estimated by averaging all the condensates sampled at *T* < 0.4*T*_c_ and *T* ≥ 0.4*T*_c_, respectively. For sequences with ϕ < 0.4, the droplet is more asymmetric and develops cavities within it for higher *T*. For sequences with ϕ > 0.4, an opposite behavior is observed, in which a larger deviation from spherical condensate is indicated for lower *T*. The different behavior of the shape anisotropy for different ϕ, illustrate the role the balance between short- and long-range interactions have on the structure of the condensates.

In order to better understand the origin of the re-entrant behavior for sequences with high hydrophobicity content, we quantified the overall geometry of the condensate by estimating its shape anisotropy (see Methods).^14^Figure 4 shows the mean shape anisotropy at low (*T* < 0.4*T*_c_) and high (*T* > 0.4*T*_c_) temperatures as a function of ϕ. For peptides with low hydrophobicity (i.e., ϕ < 0.4), the mean shape anistotropy parameter exhibits a larger deviation from 3 at higher temperature than at lower temperatures. This reflects the deviation from spherical shape droplets as the temperature increases due to breaking of intermolecular interactions. For peptides with high hydrophobicity content (i.e., ϕ > 0.4), an opposite behavior is observed in which a greater deviation for the spherical droplet (namely, larger shape anisotropy) is observed at lower temperatures. This feature, which is a manifestation of the re-entrant behavior at low *T*, can largely be explained by the emergence of short-range interactions out weighting the long-range electrostatic interactions. When short-range interactions dominate the stability of the condensate, its internal dynamics is reduced (see below), which slows the time scale for the exchange of interactions between neighboring polymers in the condensate (i.e., reduces τ_interchain_) and so lowers the probability of voids forming within the condensate.

**Figure 5:**
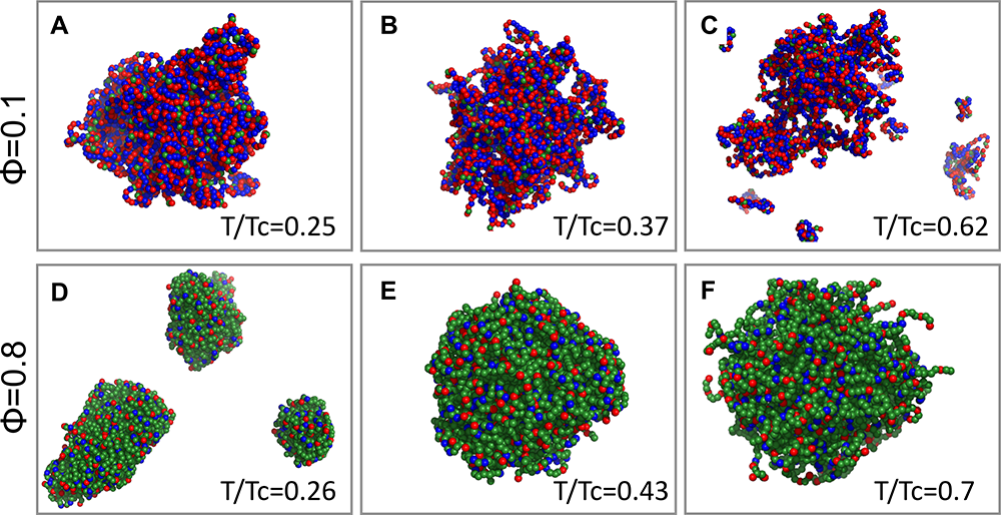
Illustration of the shape of the condensates. Snaphots of the shape of the condensates are presented for sequences with ϕ = 0.1 (A–C) and 0.8 (D–F) at three different temperatures, as indicated in each panel. Blue and red spheres correspond to positively and negatively charged residues, respectively, that participate in long-range electrostatic interactions. The green spheres correspond to hydrophobic residues that interact among themselves through short-range interactions. It is evident that, while at low *T*, near-spherical condensates are characteristic of sequences with low ϕ, fragmented condensates of elongated shapes are prevalent for sequences with high ϕ. These sequences that are rich with short-range interactions adopt a near spherical shape at relatively high temperatures comparing the sequences that are rich with long-range interactions.

To illustrate the different behaviors at low tempeartures of the condensate formed by sequences of low and high ϕ, we show snaphosts of droplets formed by sequences of ϕ = 0.1 and 0.8 at three different temperatures. Figure 5 shows that, at low temperature, the sequences of high hydrophobic content form fragmented droplets than those that are formed at higher temperature. The origin of the fragmentation of the droplet is the excess of short-range interactions whose dynamics is slow at low temperature. The formation of smaller droplets at low *T*, which may act as kinetic traps before the formation of larger condensate as the temperature increases, explains the low density phase observed only for sequences with ϕ > 0.4. For sequences with ϕ < 0.4, a large, nearly spherical, droplet is observed at low temperature, which deviates from a spherical shape as the temperature increases.

### Structural Features of the Condensate

**Figure 6:**
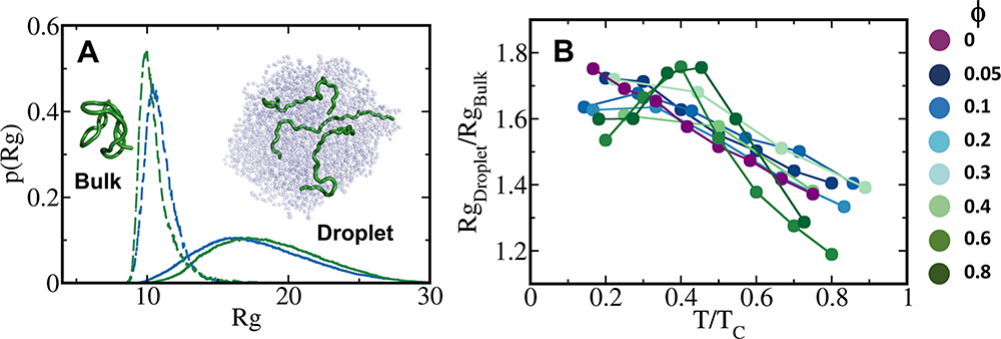
Conformational preferences of polymers in the dense phase. (A) Probability distribution of the radius of gyration (p(*R*_g_)) for polymers in the dense phase (solid lines) and in the bulk (dashed lines) at *T* = 0.5*T*_c_. The *R*_g_ distributions in the dense phase are shifted to the right and are significantly broader than in the bulk, implying the presence of more extended conformations and large-scale conformational fluctuations in the dense phase. The inset illustrates three extended conformations adopted by an IDP with ϕ = 0.4 at *T* = 0.5*T*_c_ in the dense phase, whereas a collapsed state is observed in the bulk. (B) Ratio of average polymer *R*_g_ in the dense phase to *R*_g_ in the bulk as a function of scaled temperature, *T*/*T*_c_.

The structural characteristics of the studied IDPs are very different in the dense phase compared with the bulk. The probability distribution of the *R*_g_ of IDPs in the dense phase is significantly broader in comparison with their sharp distribution in the bulk (Figure 6). The broader *R*_g_ distributions reflect the greater conformational entropy of the polymers in the condensate than in the bulk (Figure S7), as was shown recently for polyampholytes.^14^ The *R*_g_ of the polymers in the dense phase is about 1.3–1.8 times higher than in the bulk, depending on the temperature (Figure 6). As the temperature increases, the ratio *R*_g_droplet_/*R*_g_bulk_ decreases because the conformation expands more in the bulk, whereas the conformations in the condensates are less sensitive to temperature effects as they are mostly governed by the interactions they participate in with their neighbors. Consequently, the temperature dependence of *R*_g_droplet_/*R*_g_bulk_ is strong, whereas its dependence on ϕ is weaker.

In addition to the response of the IDP conformations to temperature changes or to sequence mutations, the overall shape of the condensate can be affected, too. The shape and packing of the polymers in the condensate is linearly related to the viscosity of the condensate, which in turn affects the dynamics of the dense phase. As quantified by the shape anisotropy parameter (Figure 4), a near-spherically shaped droplet is formed for sequences with ϕ < 0.4, which shows a gradual deviation from the spherical shape as the temperature increases. For sequences with ϕ > 0.4, a deviation from a spherical droplet is observed also at low temperature, which corresponds to the re-entrant behavior (see above).

### Dynamics within the Dense Phase

**Figure 7:**
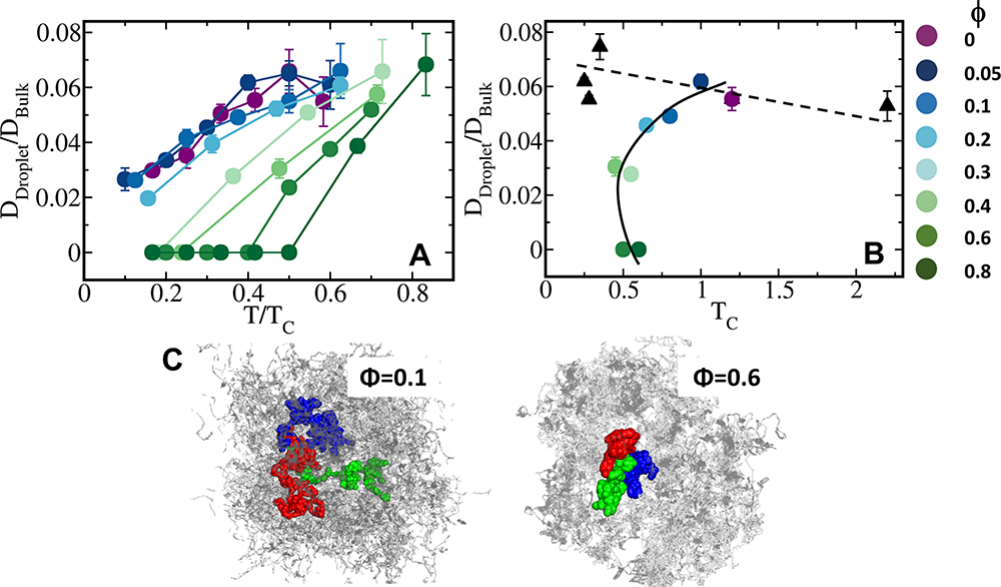
Translational diffusion in the condensate. (A) Ratio of the translational diffusion constant (*D*) in the dense droplet phase to that in the bulk (*D*_droplet_/*D*_bulk_) as a function of the temperature scaled with respect to the critical temperature, *T*/*T*_c_, for the studied sequences, colored following the color bar. (B) Correlation between *D*_droplet_/*D*_bulk_ and *T*/*T*_c_ for the eight studied systems (indicated by spheres), where the value of ϕ is 0–0.8 (see color bar key). For comparison, corresponding data concerning five 40-residue polyampholytes were added (black triangles, adopted from ref,^14^ (see Figure S2)). In this plot, the *D*_droplet_/*D*_bulk_ ratios were calculated at *T* = 0.4*T*_c_. The five sequences of polyampholytes each comprise 20 positively and 20 negatively charged residues, and differ in their charge organization pattern, which is quantified by the κ value. We note that the polyampholyte with κ = 0.55 is identical to the sequence with ϕ = 0. The solid and dashed lines are intended to guide the eye. (C) A pictorial illustration of translational diffusion in the condensate phase for systems with ϕ = 0.1 and 0.6 at *T* = 0.4*T*_c_. These plots show projections of the center of mass of three independent polymers (colored green, red, and blue) for 2000 timesteps, whereas the centers of mass of the other chains in the droplet are colored gray. The plots illustrate that an increase in ϕ slows down diffusion in the dense phase. At ϕ = 0.6, there is no such diffusion of polymers, even at *T*/*T*_c_ = 0.4 in the dense phase, which indicates an increased possibility of gel-like assembly or solid like behavior as the short-range interaction content increases in a sequence.

To investigate the dynamics of peptides in the dense phase, we calculated their translational diffusion constant in the dense phase and in the bulk. This was achieved by estimating the slope of the mean squared displacement (MSD) of the centers of mass (COM) of the polymers, which in three dimensions satisfies the equation *D* = ⟨(*x*(*t*) – *x*(0))^2^⟩ + ⟨(*y*(*t*) – *y*(0))^2^⟩ + ⟨(*z*(*t*) – *z*(0))^2^⟩/6*t*. When calculating the MSD of the polymers in the droplet, we ensured that they remained in the dense phase for the entire calculation time-window (0 to *t*). We also removed the effect of the COM motion of the droplet on the MSD of the polymers in the dense phase by subtracting the average MSD of the droplet COM from the average MSD of the polymers in the droplet. For a convenient comparison to experimental data, we show the ratio *D*_droplet_/*D*_bulk_ against *T*/*T*_c_ for the sequences studied. For a fixed value of *T*/*T*_c_, the *D*_droplet_/*D*_bulk_ ratio tends to decrease 2–5 times, depending on ϕ, indicating that diffusion in the droplet is 15–50 times slower than in the bulk. As ϕ increases, diffusion in the droplet slows down. For ϕ > 0.3, no diffusion within the droplet is observed at low temperatures. This indicates that short-range interactions reduce dynamics in the dense phase and that the condensate does not exhibit liquid properties, particularly at low temperatures. For example, for the sequence with ϕ = 0.6, diffusion is observed only for *T*/*T*_c_ > 0.4. For the sequence with ϕ = 0.8, liquid behavior of the condensate is detected only for *T*/*T*_c_ > 0.5 (Figure 7A). This phenomenon can also be attributed to the loss of electrostatic interactions. Short-range LJ and electrostatics interactions have very different natures. When electrostatic interactions are largely present in a condensate, they exert a long-range attractive pull or repulsion on a polymer in the condensate resulting in increased dynamics and liquid-like mobility in condensates. However, the prevalence of short-range LJ interactions in the condensates lack that long-range feature, such that they exert an influence only within the close vicinity of the polymers. Such short-range hydrophobic interactions break more slowly than long-range electrostatic interactions, so leading to relatively slow diffusivity.

The decrease in *D* as ϕ increases suggests that long-range electrostatic interactions play a role in the liquid behavior of the condensate. Diffusion, however, may also depend on the stability of the condensate. To address this question, we examined the relationship between *D*_droplet_/*D*_bulk_ and *T*_c_ (Figure 7B). Indeed, the main observation from Figure 7 is that polyampholytes exhibit greater liquid properties in the droplet phase and that the introduction of hydrophobic residues (i.e., gradual increase in ϕ) results in a significant decrease in the diffusion coefficient. Furthermore, a complex relationship between the diffusion coefficient and *T*_c_ is revealed. For polyampholytes, mixing of the charges (defined by κ, Figure S8), which corresponds to lower *T*_c_, is slightly anticorrelated with the *D*_droplet_/*D*_bulk_ ratio. However, a gradual increase in ϕ (achieved by mutating charged residues to hydrophobic ones), which results in a decrease in *T*_c_, is coupled with a decrease in *D*_droplet_/*D*_bulk_. Further increasing φ results in stabilization, which is reflected by higher *T*_c_ (see also Figure 1), but *D*_droplet_/*D*_bulk_ continues to decrease. The liquid-like nature of the dense phase partially arises from the electrostatic interactions. The movement of polymers in the dense phase is driven by a long-range repulsions and attractive pulls from other polymers. As we increasingly mutate the electrostatic residues, which exert a short-range attractive potential, we observe a significant decrease in polymer diffusion in the dense phase as a result of stabilizing short-range contacts formed between neighboring polyampholytes. Figure 7C shows the trajectory view of the COM of three selected polymers at *T* = 0.4*T*_c_ for two different sequences, namely, ϕ = 0.1 and 0.6. For ϕ = 0.6, we observe a localized dynamic, whereas liquid-like diffusivity is pronounced in the scenario of ϕ = 0.1.

**Figure 8:**
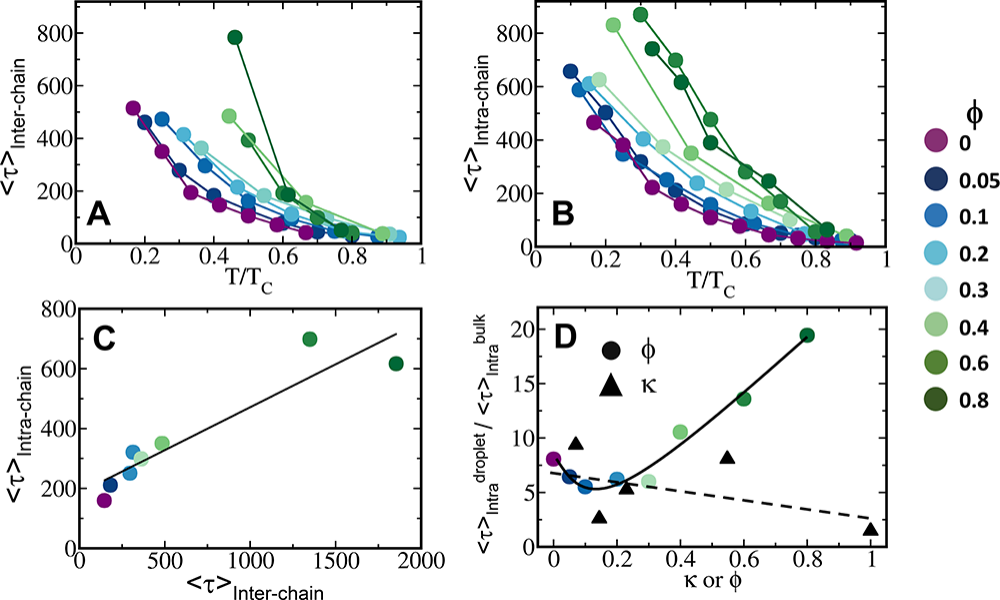
Dynamics of inter- and intramolecular interactions in the condensates. (A) Averaged time scales for the nearest neighbor exchange dynamics of polymers in the dense phase (interchain dynamics) as a function of temperature (*T*) below the critical temperature (*T*_c_) of the respective sequences. For a given value of *T*/*T*_c_, the plots show a gradual increase in the time scale of the dense phase as the ϕ parameter of the sequence increases (see color bar). (B) Averaged time scales of correlated end-to-end fluctuation dynamics (intrachain dynamics)) for polymers in the condensate and bulk as a function of temperature, below the respective critical temperatures of the sequences. (C) Correlation between the time scales of inter- and intrachain dynamics. The presented time scales are for *T* = 0.4*T*_c_. (D) Ratio of the intrachain dynamics in the condensate versus the bulk for the eight sequences for varying ϕ values (circles) and for polyampholytes (black triangles). The polyampholytes are fully charged (i.e., ϕ = 0) and differ in their charge distribution (defined by κ). The solid and dashed lines are intended to guide the eye.

The translational diffusion of a polymer in the dense phase is inherently related to the formation and breakage of the interchain contacts it forms with neighboring polymers as well as to its own intrachain dynamics. To probe the molecular origin of the reduced diffusion coefficient as ϕ increases, we studied the kinetics of exchange of nearest neighbor polymers around a central polymer (designated by τ_interchain_) in the dense phase. In addition, we estimated the kinetics of the conformational dynamics within each polymer in the dense phase by estimating the time scale of relaxation of the end-to-end distance (designated by τ_intrachain_). As the temperature increases toward criticality, one observes faster dynamics for all the systems with respect to both inter- and intramolecular dynamics. For the same value of *T*/*T*_c_, both interchain dynamics (Figure 8A) and intrachain dynamics (Figure 8B) slow down 2–4-fold as ϕ increases, thus, suggesting that short-range interactions are responsible for the slower dynamics. Moreover, these two time scales are correlated, although their correlation deviates from linearity (Figure 8C). In particular, for ϕ > 0.6, the value of τ_interchain_ increases to a greater extent than that of τ_intrachain_, which may explain the nonliquid behavior of the condensate in the presence of a high fraction of short-range interactions.

It is interesting to note that the end-to-end distance fluctuation dynamics is 4–20 times slower in the dense phase than the bulk, depending on temperature and ϕ (Figure 8D). In order to understand the effect of the short-range interactions on the internal dynamics of the polymers, we added the corresponding values for the end-to-end fluctuation dynamics in the dense phase and bulk of polyampholyte sequences (i.e., ϕ = 0) to the figure (Figure 8D). We observe no clear correlation between the time scales of the four polyampholytes. Their end-to-end dynamics in the condensate relative to that in bulk is similar to sequences with ϕ < 0.4, which indicates that their conformational dynamics is no more than 10 times slower in the dense phase compared with the bulk. However, for sequences with ϕ > 0.4, the conformational dynamics in the condensate is even 20 times slower than in the bulk.

### Dependence of Short-Range Interaction Strength on Stability and Dynamics

**Figure 9:**
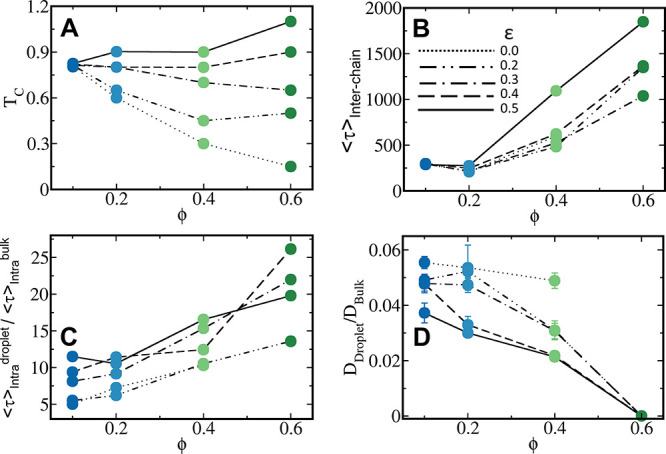
Dependence of short-range interaction strength on stability and dynamics. (A) Critical temperature (*T*_c_) for specific sequences, with ϕ = 0.1, 0.2, 0.4, and 0.6 for dispersion interaction strengths (ε) 0.0, 0.2, 0.3, 0.4, and 0.5 at *T*/*T*_c_ = 0.4. As the contribution of the dispersion interactions increases in the condensate, the dense phase tends to stabilize at a higher critical temperature. The effect of interaction strength is minimal for ϕ = 0.1. (B) Averaged time scale of nearest neighbor exchange dynamics as a function of ϕ for different values of ε at *T*/*T*_c_ = 0.4 for the specified sequences. (C) Ratio of the time scales for end-to-end fluctuation dynamics in the condensate vs the bulk as a function of ϕ for differnet values of ε at *T*/*T*_c_ = 0.4 for the specified sequences. (D) Ratio of translational diffusion constant (*D*) in the dense phase vs the bulk as a function of ϕ for different values of ε at *T*/*T*_c_ = 0.4. The color scheme of the sequences is as per Figure 1.

Our discussion has so far focused on the effect of the hydrophobic fraction, which engages in short-range interactions, on the dynamics and stability of IDP condensates. We now ask how the condensates’ characteristics are affected by the energetic strength of these short-range interactions (ε), which may be increased by mutating the sequence to include more hydrophobic residues. To understand how short-range interaction strength affects the stability of the condensate, we calculated the phase diagram for varying ε (0–0.5). Up to ϕ = 0.2, a small change in stability is observed, whereas from about ϕ = 0.2–0.4 and upward, we observe a prominent increase in the stability of the condensate as the interaction strength increases, as indicated by the increase in the *T*_c_ of the sequences (Figure 9A). The short-range interaction energy tends to depend on ε only at higher ϕ values, leading to a distinct and significant stabilization. Although we observe a significant enhancement in the time scale of inter- and intrachain dynamics at higher ϕ because of an enhancement in ε (Figure 9B,C), the effect on global diffusion is small in such condensates. Rather, we observe a significant drop in the *D*_droplet_/*D*_bulk_ ratio when ϕ = 0.1–0.2, under which conditions the dense phase is still liquid-like with the presence of a higher charge content (Figures S9–S11). Perturbation due to the enhanced strength of the mutated short-range sites seems to have a larger effect under these conditions.

## Conclusions

The sequence–structure–function relationship holds not only for globular proteins, but also for IDPs that form condensates via LLPS. Many studies have endeavored to address how the sequence of the IDP affects the properties of the condensates. Electrostatic, π–π, and cation−π interactions are highlighted to be among the main driving forces for LLPS. Clearly, LLPS depends not only on the proportions of charged, hydrophobic, and aromatic residues in IDPs, but also on their organization and segregation. The pattern of hydrophobic^27,30^ or charged^14,28,61^ residue organization has been shown to have a strong effect on LLPS.

In this study, we focused on the interplay between hydrophobic and charged residues by investigating the condensate formed by a series of IDPs possessing varying hydrophobic residue fractions in the range 0–0.8 (i.e., their charged residue fraction was 0.2–1). These sequences, while having a net charge of zero, differed in the amount of short- and long-range interactions they could engage in, which significantly affected the stability and internal dynamics of the condensates. Replacing charged residues with hydrophobic residues has a more pronounced biophysical effect on LLPS than altering the charge pattern along the sequence.^14^ The pattern of hydrophobic residues is also expected to affect LLPS,^30,62^ but this aspect was not studied systematically in the current study. We found that replacing long-range interactions with short-range ones can decrease the critical temperature by more than 50%. The decrease in the stability of the droplet is coupled with a decrease in the translational diffusion of each IDP in the dense phase. Changes in the liquid properties of the condensate with increased short-range interactions are linked to a slower breaking of intermolecular contacts with neighboring chains in the droplet, which is coupled with slower configurational chain dynamics. The effect of IDP hydrophobicity on condensate stability and dynamics is not monotonic. For a very high fraction of hydrophobic residues (i.e., a large number of short-range interactions), the critical temperature of LLPS increases and the phase diagram shows re-entrant behavior, which indicates ordered states, consistent with the observation of very sluggish diffusion and very slow intra- and intermolecular dynamics. This indicates the role long-range electrostatic interactions have in maintaining the liquid properties of the condensates and that the balance between short- and long-range interactions, as implied by the sequence composition and organization, can affect their structure, stability, and dynamics.

## Supporting Information Available

Paramaterization of the short-range potential; Potential of short- and long-range interactions; Convergence of the simulations; Size-effect of the phase diagrams; Enthalpic stability of the condensate; Conformational entropy; Effect of the short-range potential strength on phase diagram, enthalpy, and diffusion (PDF)
