# Supplementary material for: Biophysics of Phase Separation of Disordered Proteins Is Governed by Balance between Short- And Long-Range Interactions
Source: J Phys Chem B. 2021 Feb 25;125(9):2202–11. doi: 10.1021/acs.jpcb.0c09975 (PMC8028311; doi:10.1021/acs.jpcb.0c09975)
Supplement: Supplementary file 1 — jp0c09975_si_001.pdf [file jp0c09975_si_001.pdf]

Supporting Information

Biophysics of Protein Condensates is Governed by Balance between Short- and Long-range Interactions

Milan Kumar Hazra and Yaakov Levy

Department of Structural Biology, Weizmann Institute of Science, Rehovot 76100, Israel

Proteins used to benchmark the short-range interaction strength (ε)

CspTm
GPGMRGKVKW FDSKKGYGFI TKDEGGDVFV HWSAIEMEGF KTLKEGQVVE FEIQEGKKGG
QAAHVKV

IN
GSHCFLDGID KAQEEHEKYH SNWRAMASDF NLPPVVAKEI VASCDKCQLK GEAMHGQVDC

ProTα-N
GPSDAAVDTS SEITTKDLKE KKEVVEEAEN GRDAPANGNA ENEENGEQEA DNEVDEECEE
GGEEEEEEEE GDGEEEDGDE DEEAESATGK RAAEDDEDDD VDTKKQKTDE DD

ProTα-C
MAHHHHHHSA ALEVLFQGPM SDAAVDTSSE ITTKDLKEKK EVVEEAENGR DAPANGNANE
ENGEQEADNE VDEECEEGGE EEEEEEEGDG EEEDGDEDEE AESATGKRAA EDDEDDDVDT
KKQKTDEDD

R15
KLKEANKQQN FNTGIKDFDF WLSEVEALLA SEDYGKDLAS VNNLLKKHQL LEADISAHED
RLKDLNSQAD SLMTSSAFDT SQVKDKRETI NGRFQRIKSM AAARRAKLNE SHRL

R17
RLEESLEYQQ FVANVEEEEA WINEKMTLVA SEDYGDTLAA IQGLLKKHEA FETDFTVHKD
RVNDVAANGE DLIKKNNHHV ENITAKMKGL KGKVSDLEKA

hCyp
SSFHRIIPGF MSQGGDFTRH NGTGGKSIYG EKFEDENFIL KHTGPGILSM ANAGPNTNGS
QFFISTAKTE FLDGKHVVFG KVKEGMNIVE AMERFGSRNG KTSKKITIAD SGQLE

Protein L
MEEVTIKANL IFANGSTQTA EFKGTFEKAT SEAYAYADTL KKDNGEWTVD VADKGYTLNI
KFAG

ACTR
GTQNRPLLRN SLDDLVGPPS NLEGQSDERA LLDQLHTLLS NTDATGLEEI DRALGIPELV
NQGQALEPKQ D

hNHE1cdt
MVPAHKLDSP TMSRARIGSD PLAYEPKEDL PVITIDPASP QSPESVDLVN EELKGKVLGL
SRDPAKVAEE DEDDDGGIMM RSKETSSPGT DDVFTPAPSD SPSSQRIQRC LSDPGPHPEP
GEGEPFFPKG Q

sNase

ATSTKKLHKE PATLIKAIDG DTVKLMYKGQ PMTFRLLLVD TPETKHPKKG VEKYGPEASA
FTKKMVENAK KIEVEFDKGQ RTDKYGRGLA YIYADGKMVN EALVRQGLAK VAYVYKPNNT
HEQHLRKSEA QAKKEK

α-synuclein

MDVFMKGLSK AKEGVVAAAE KTKQGVAEAA GKTKEGVLYV GSKTKEGVVH GVATVAEKTK
EQVTNVGGAV VTGVTAVAQK TVEGAGSIAA ATGFVKKDQL GKNEEGAPQE GILEDMPVDP
DNEAYEMPSE EGYQDYEPEA

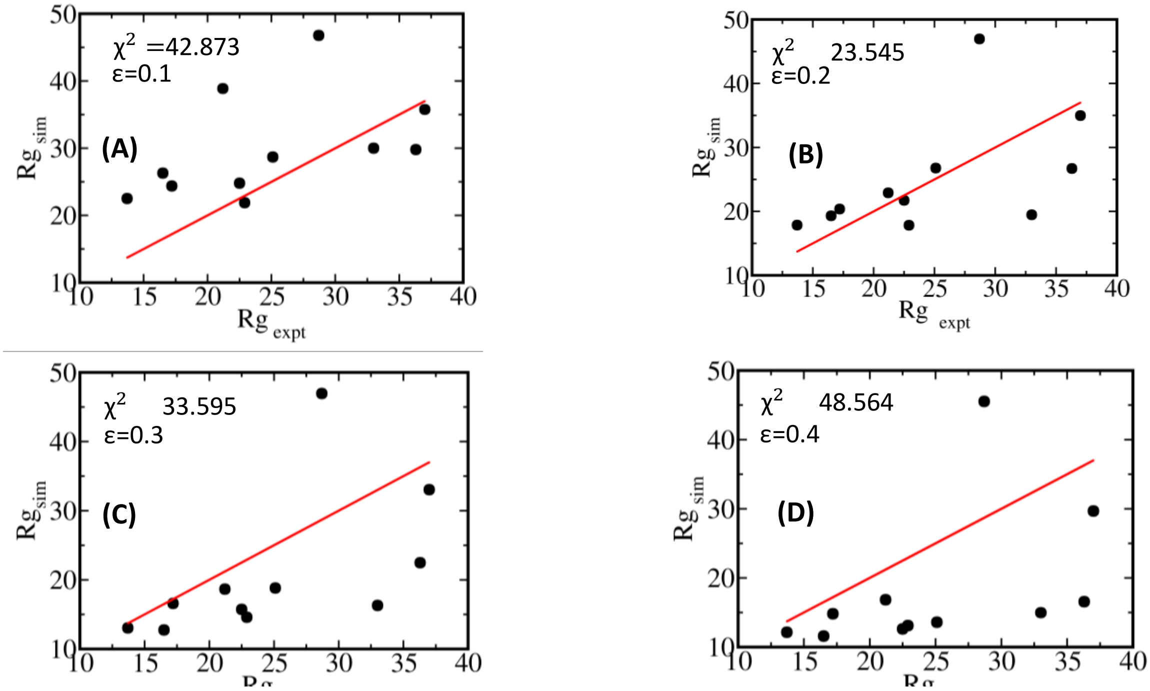

Figure S1. Comparison of radius of gyration (Rg) between simulations and experiments for different ε parameter of short-range interaction. The list of the proteins and their experimental Rg was obtained from literature and can be found elsewhere[1]. The deviations χ^2 between simulations and experiments are shown in the top left corner of the panel. χ^2 seems to have minimum value for ε=0.2 which corresponds to highest similarity to the experimental values.

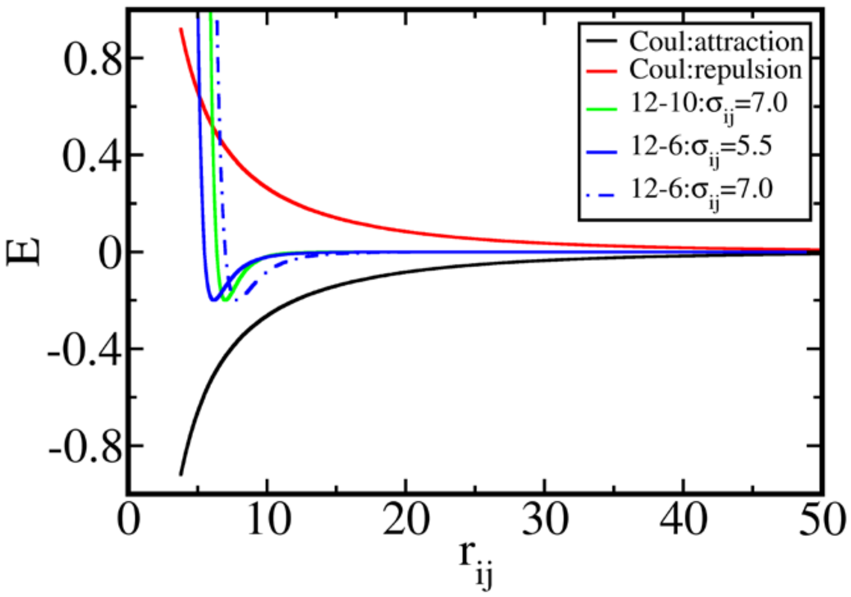

Figure S2. Comparison between the short- and long-range potentials. The 10-12 and 10-6 Lennard-Jones potentials that represent short-range interactions and the Debye-Huckel Coulombic potential that represnts the long-range interactions are plotted as a function of the pairwise deistance, r_{ij}. The 12-10 Lennard-Jones is plotted for optimal distance of 7Å and the 12-6 potentail is plotted for both 5.5 and 7.0Å. The Debye-Huckel is plotted for both repulsive and attrcative case for salt concnetration of 0.02M. The plot illustartes the differnce between the Lennard_Jones and the Columbic potentials and that the differnces between the 12-10 and 12-6 are much smaller.

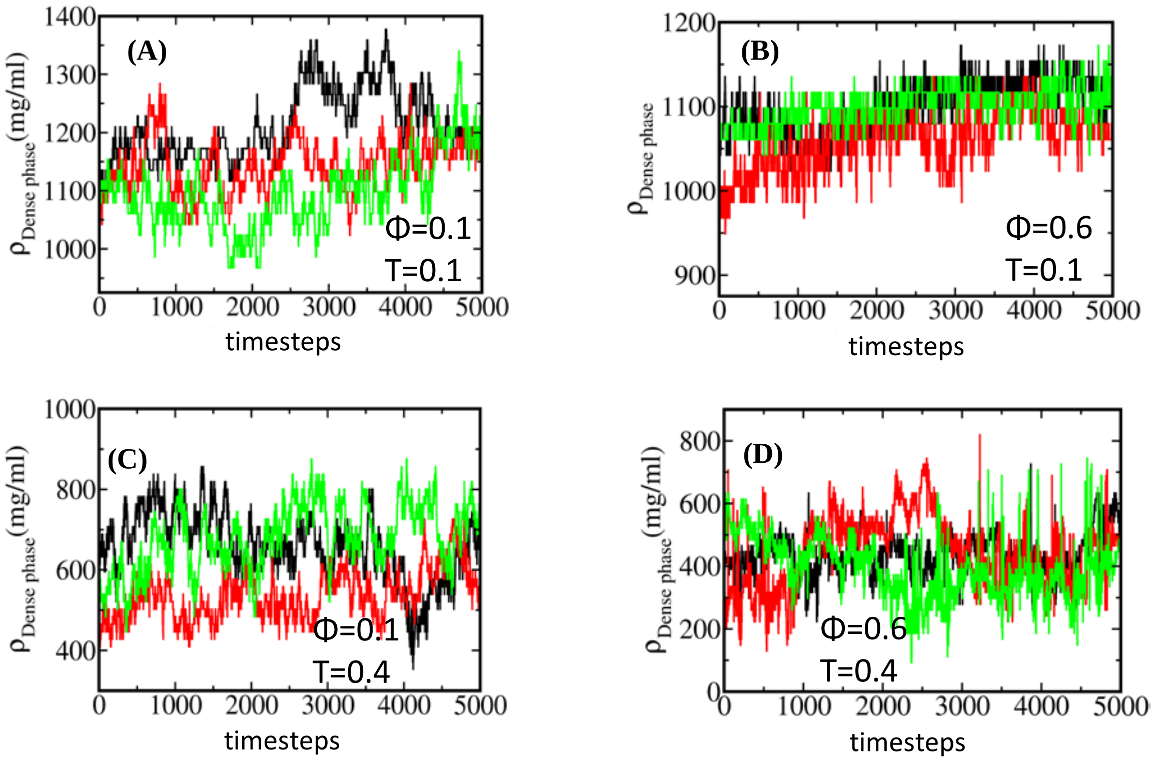

Figure S3. Convergence of the coarse-grained simulations for condensate formation. The time evolution of the density of the dense phase of sequences with ϕ=0.1 and 0.6 is plotted at two temperatures (T=0.1 and 0.4). For each case, three trajectories are shown designated in red, green and black.

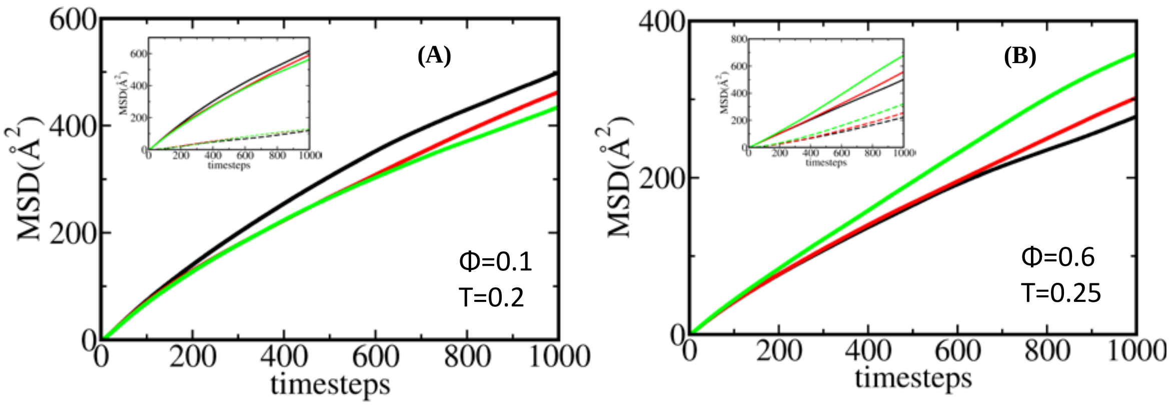

Figure S4. Convergence of the coarse-grained simulations for condensate formation. The MSD of the chain in the droplet (solid lines) and of the center-of-mass of the droplet (dashed lines) are shown for two sequences with ϕ=0.1 and 0.6. For each case, three simulations are shown (in green, red and black).

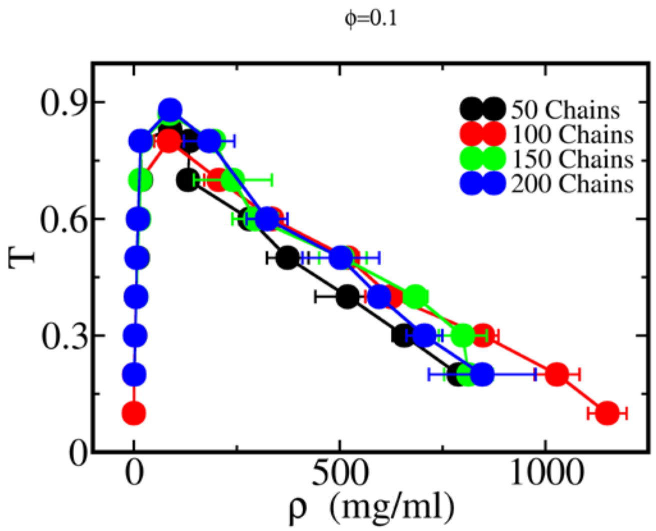

Figure S5. Effect of the number of chain on the phase diagram of LLPS. The size effect was examined by studying the LLPS of 50, 100, 150, and 200 chains of sequences of ϕ=0.1. The phase diagrams are highly similar regardless of the size of the systems.

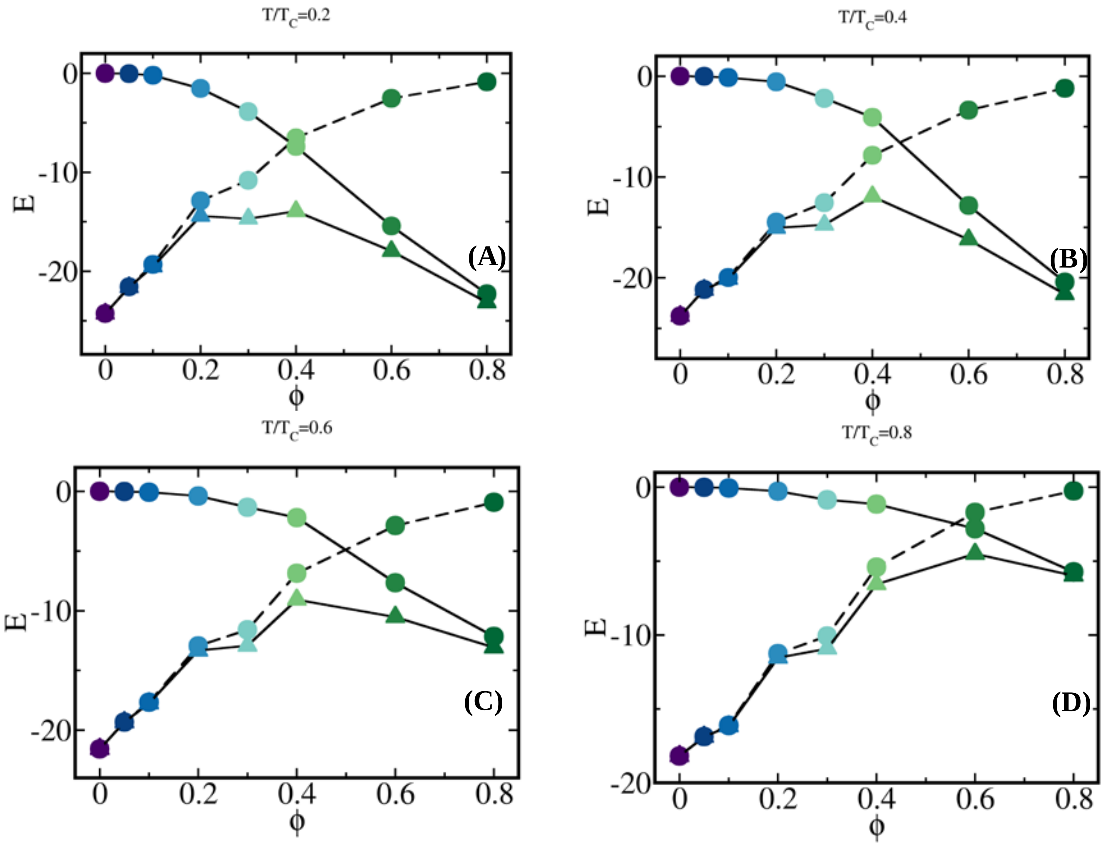

Figure S6. Enthalpic stability of the dense phase. Trend in total inter-chain energy (*i.e.*, the sum of the short- and long-range energies, shown with triangles), in the dense phase as a function of ϕ at T=0.2T_C, 0.4T_C, 0.6T_C, and 0.8T_C. The inter-chain short- and long-range interaction energy are shown with solid and dashed lines, respectievly. Similar to Figure 5C.

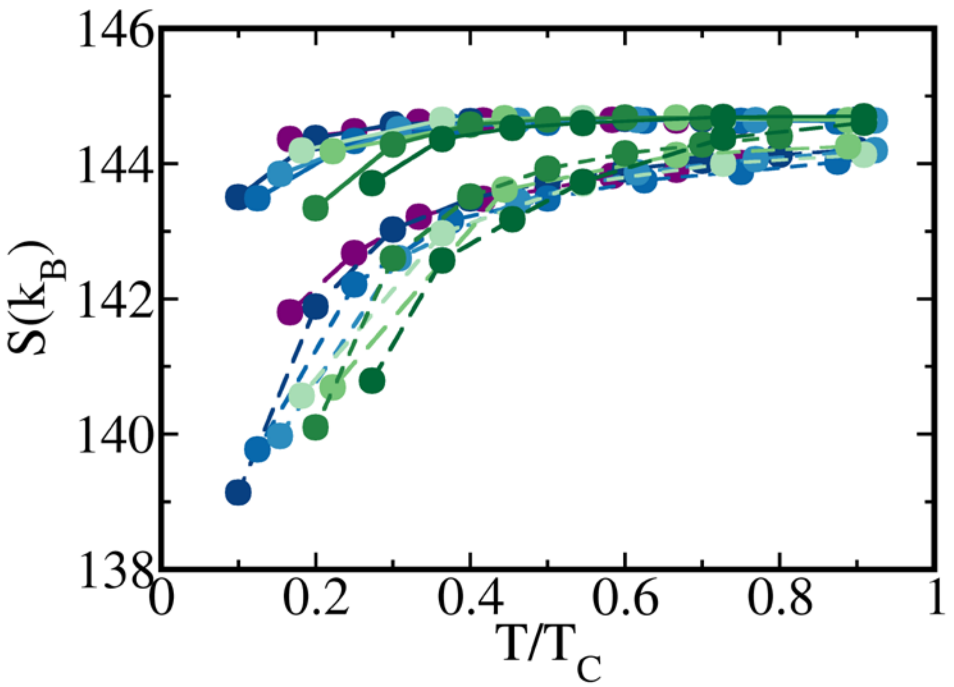

Figure S7. Conformational entropy in dense phase and bulk for the sequences with various ϕ. Conformational entropy is estimated from variations in all the dihedral angles within the polymer, as follows, $S = \sum_{i=1}^{n_{polymer}} \sum_{j=1}^{n_{dihedral}-3} \sum_{k=-\pi}^{\pi} p_k^j \ln(p_k^j)$, where p_k^j represents the probability of the jth dihedral angle to sample the kth conformation. Conformational entropy in dense phase is higher than bulk and less sensitive toward the variation of ϕ. Higher conformational entropy in dense phase is also in accordance with the extended conformation of polymers in droplet as well as a broad distribution for radius of gyration (Rg) in dense phase signifying intense conformal fluctuation.

Sequence	κ
KKKKKKKKKKKKKKKKKKKKEEEEEEEEEEEEEEEEEEEE	1.0
EEEEEEEEEKEKEEKKKKKKKKKKKKKKKKEEEKEEKEEE	0.55
EEKEEEEEKEEKEKEKKEEKEKEKKEEKKKKKKKKKKEEE	0.23
KKEKKEKKEKKEKEKEKKEEKKKEKKEEEEEEEEKEKKEE	0.14
EKKKKKKEEKKEEEEKKEEKKEKKEEKEKEEKKEKKEEEE	0.07

Figure S8. Fully charged sequences (ϕ=0) with their charge mixing parameter (κ) used in this study. Adopted from PCCP, 22, 19368-19375, (2020)

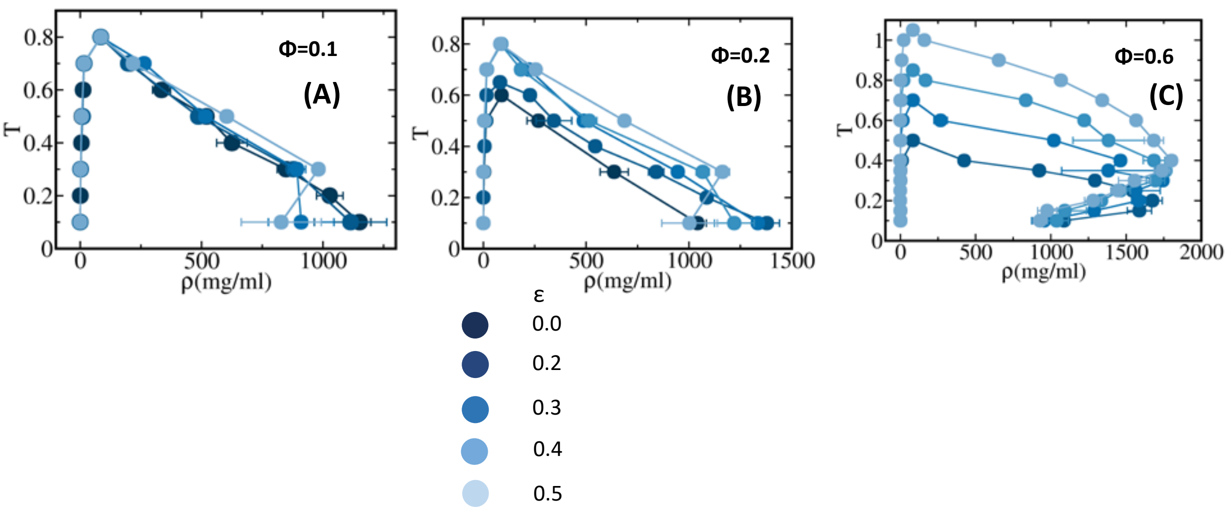

Figure S9. Short-range interaction strength (ε) dependence of phase diagram for specific sequences with ϕ=0.1, 0.2 and 0.6. Critical temperature is insensitive to the variation of short-range interaction strength for ϕ=0.1, 0.2 but increases significantly at ϕ=0.6 as one tends to increase ε as short-range interactions mediates the stability of the droplet. For ϕ=0.6 and ε=0, we don't see any stable phase separated droplet. Colour panel denotes the interaction strength.

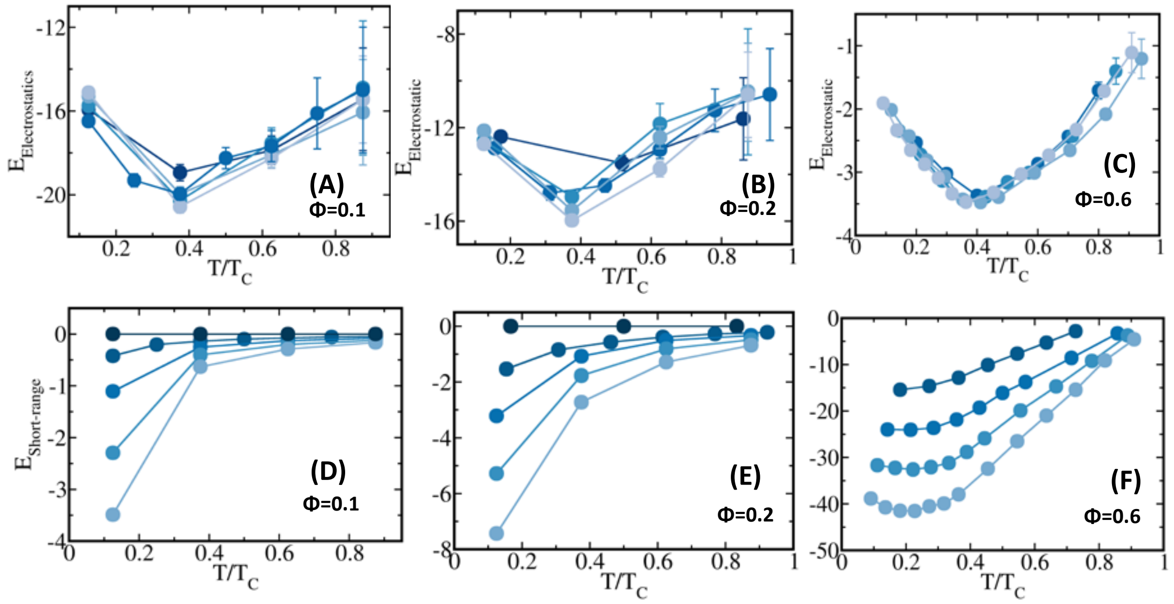

Figure S10. Inter-chain enthalpic contribution to the stability. Panel (A-C) denotes the inter-chain electrostatic contribution to the energetics in condensate for different short-range interaction strength (ε) as a function of T/T_c. As expected, long range electrostatic energy is invariant to the ε-variation. Panel (D-F) denotes the inter-chain short-range dispersion energy as a function of T/T_c in condensate as ε varies. The effect of interaction strength is minimal for ϕ=0.1 but as ϕ increases one observes a significant stabilization due to an enhancement of the strength of each inter-chain contact. Color scheme is similar to the previous figure.

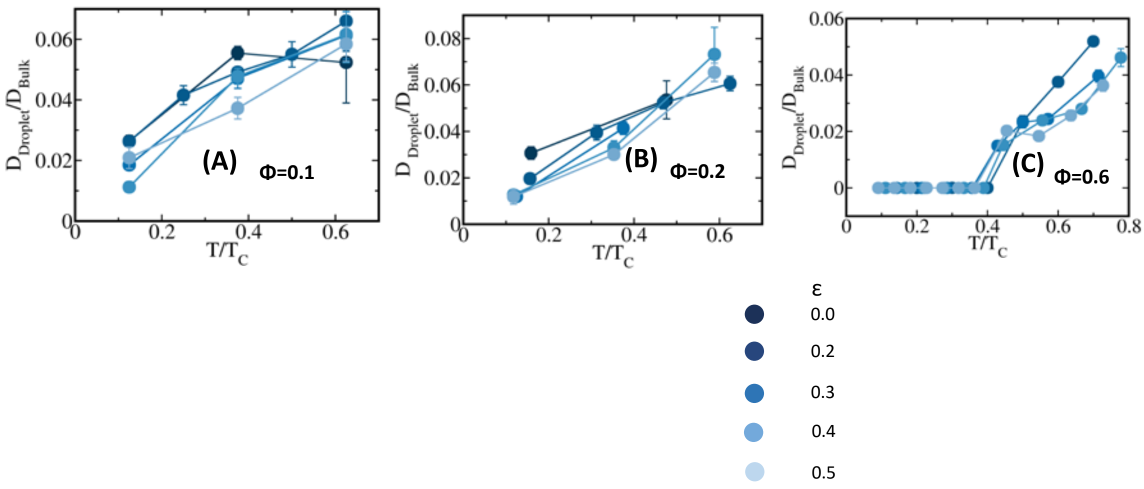

Figure S11. Translation diffusion as a function of short-range interaction strength. Ratio of the translational diffusion constant (D) in the dense droplet phase to that in the bulk ($D_{Droplet}/D_{Bulk}$) as a function of the temperature scaled with respect to the critical temperature, T/T_C, for the studied sequences with ϕ=0.1, 0.2 and 0.6. we observe a significant dropdown in the ratio $\frac{DDroplet}{DBulk}$ in the regime ϕ=0.1- 0.2, where the dense phase is still liquid like with the presence of higher charge content and the perturbation due to enhanced strength of the short-range interaction seems to have a larger effect in this regime. For ϕ=0.6, the diffusion of polymers in dense phase at sufficiently low T is completely absent (ε=0 and ϕ=0.6, there is no-such stable phase separation).

References

1) Dignon, G. L.; Zheng, W.; Kim, Y. C.; Best, R. B.; Mittal, J. Sequence Determinants of Protein Phase Behavior from a Coarse-Grained Model. *PLoS Computational Biology* **2018**, *14* (1). https://doi.org/10.1371/journal.pcbi.1005941.
